# The Community Acute Respiratory Infection surveillance programme: an evaluation of a newly established surveillance programme in Scotland

**DOI:** 10.1093/eurpub/ckae200

**Published:** 2024-11-28

**Authors:** Tonje S Laird, Mark Hamilton, Catriona Oliver, Fatima Sadiq, Yomna Moawad, Josie Evans

**Affiliations:** Respiratory Team, Clinical and Protecting Health Directorate, Public Health Scotland, Glasgow, United Kingdom; Respiratory Team, Clinical and Protecting Health Directorate, Public Health Scotland, Glasgow, United Kingdom; Respiratory Team, Clinical and Protecting Health Directorate, Public Health Scotland, Glasgow, United Kingdom; Respiratory Team, Clinical and Protecting Health Directorate, Public Health Scotland, Glasgow, United Kingdom; Respiratory Team, Clinical and Protecting Health Directorate, Public Health Scotland, Glasgow, United Kingdom; Respiratory Team, Clinical and Protecting Health Directorate, Public Health Scotland, Glasgow, United Kingdom

## Abstract

The Community Acute Respiratory Infection (CARI) surveillance programme, established by Public Health Scotland (PHS) in November 2021, aims to monitor respiratory infections in communities, replacing prior schemes to ensure early detection of outbreaks and inform public health interventions. Positioned as a cornerstone of PHS’s national infectious respiratory diseases plan, CARI is pivotal for safeguarding public health. This study presents key findings from the 2022/23 CARI season and evaluates the programme’s performance during this period. CARI uses a network of sentinel general practitioner (GP) practices across Scotland to monitor patients with acute respiratory infection symptoms, employing multiplex polymerase chain reaction testing for 10 common pathogens. Results are linked to enhanced surveillance data, providing insights into infection trends during the season. The evaluation comprised an online GP survey and a quantitative assessment of programme performance. In the 2022/23 season, 180 GP practices participated in CARI, testing 15,823 samples. Swab positivity peaked in December 2022, driven by a large spike in influenza A activity. The evaluation showed that CARI is highly useful, with positive feedback on simplicity, flexibility, and acceptability. Representativeness varied across health boards and age groups. Despite occasional laboratory processing delays, data quality remained good, with timely reporting and stable participation. CARI reflected patterns in infections observed in secondary care in Scotland and Europe, providing valuable insights into disease patterns and impact. It also provided timely intelligence to key decision-makers, enabling prompt public health response. Changes for the 2023/24 season aim to further optimize the programme.

## Introduction

In its updated guidance on severe acute respiratory syndrome coronavirus 2 (SARS-CoV-2) and influenza surveillance, the World Health Organization (WHO) stressed the importance of monitoring influenza and other respiratory pathogens alongside coronavirus disease 2019 (COVID-19) using an integrated approach [1]. This draws on insights from countries that adapted influenza surveillance during the pandemic. The guidance highlights strategies to improve sentinel surveillance systems, which collect detailed data at selected sites, ensuring resilience for global and national surveillance. Global general practitioner (GP) sentinel networks, like those identified in the Sentiworld mapping project [2], play a key role, contributing essential data on respiratory diseases to strengthen integrated surveillance efforts.

Public Health Scotland (PHS) responded to WHO’s guidance by launching the Community Acute Respiratory Infection (CARI) surveillance programme in November 2021 [3]. The CARI programme replaced the previous COVID-19 surveillance programme, which succeeded the pre-COVID-19 GP sentinel scheme for influenza and other pathogens. The programme’s objective is to monitor the burden and impact of acute respiratory infection (ARI) in the community, supporting public health actions. Now central to PHS’ national infectious respiratory diseases plan, CARI operates year-round, ensuring early detection of community transmission, identifying high-risk groups, and detecting outbreaks.

This paper presents key findings from the 2022/23 CARI surveillance season and evaluates the programme’s performance.

## Methods

**Figure 1.**
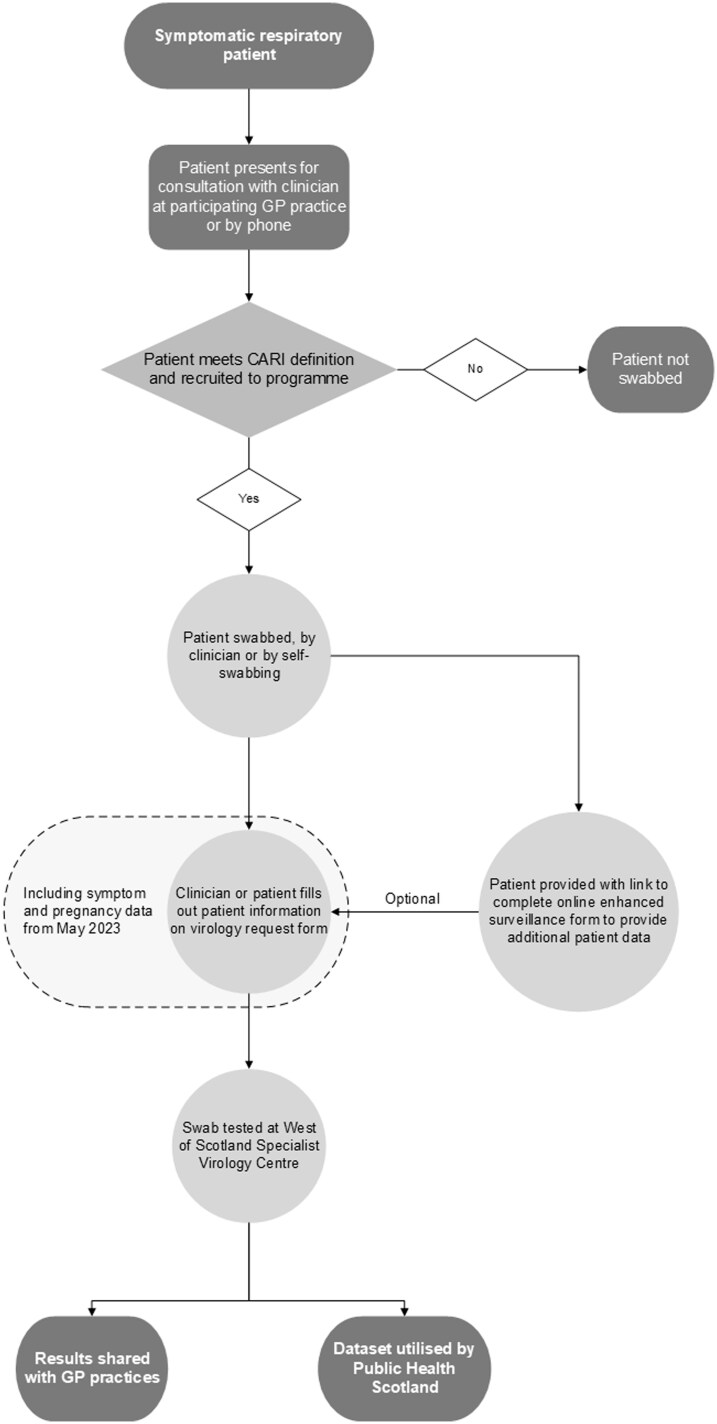
The patient pathway in the CARI surveillance programme.

The CARI programme engages a network of sentinel GP practices across Scotland to recruit patients exhibiting ARI symptoms. The case definition is a sudden onset of at least one of four symptoms (cough, sore throat, shortness of breath, coryza) diagnosed by clinicians within 7 days [4]. Clinicians verbally consent and recruit patients based on their judgement, with no set recruitment threshold. Figure 1 shows the patient pathway, where swabbing occurs during consultations or through self-swabbing kits provided to patients. An online link allows clinicians or patients to complete an enhanced surveillance form (ESF), capturing data on 16 symptoms, travel history, and pregnancy status. The ESF can be filled out by either party, depending on local practice. Data are linked to sample results using the community health index (CHI) number. GP practices receive £15 per testable sample. Full CARI methods are detailed in the programme protocol [5].

Samples from CARI patients are sent to the laboratory for multiplex polymerase chain reaction testing of 10 respiratory pathogens, including influenza A (with sub-typing), influenza B (with lineage determination), SARS-CoV-2, respiratory syncytial virus (RSV), adenovirus, coronavirus (non-SARS-CoV-2), human metapneumovirus, rhinovirus, parainfluenza, and *Mycoplasma pneumoniae*. Results are sent to GPs, and swab positivity estimates are calculated to monitor levels and trends of respiratory infections in the community. Whole genome sequencing for SARS-CoV-2 helps detect new variants. PHS links positive results to enhanced surveillance data, and also data on hospital admissions and mortality from other surveillance sources, enabling comparisons across groups. This generates a comprehensive understanding of respiratory disease trends and impact at population level. These data inform health protection teams, the Scottish Government, and key stakeholders. GP practices receive monthly reports, and weekly data are publicly shared [6].

### Evaluation of the CARI programme

The evaluation for the 2022/23 season used the European Centre for Disease Prevention and Control (ECDC) and Centers for Disease Control and Prevention (CDC) guidelines as a framework for evaluating a surveillance system [7, 8]. Data analyses were performed on CARI data collected between 3 October 2022 (ISO week 40) and 1 October 2023 (ISO week 39).

#### GP survey

In November 2023, following the 2022/23 season, an online survey assessed stakeholders’ experiences with CARI, focusing on interaction with the CARI team and programme processes. The survey was sent to the main contact at each of 143 participating practices, with a request to share it with other staff. The exact number of recipients was therefore unknown.

#### Simplicity

A surveillance programme’s simplicity refers to its structure and how easy its processes are for end-users [7, 8]. Evaluation of simplicity was based on GP survey feedback and observed data management processes.

#### Flexibility

A flexible surveillance programme adapts to changing needs or conditions [7, 8]. This was assessed through observed changes and GP survey responses.

#### Acceptability

Acceptability reflects the willingness of individuals and organizations to participate in the surveillance programme [7, 8]. Recruitment, withdrawal rates, and GP survey feedback were used to evaluate this, but patient and or other stakeholder acceptability data were unavailable.

#### Representativeness

A representative surveillance programme accurately describes a health event’s occurrence and distribution by place and person [7, 8]. Geographical and demographic representativeness was assessed by comparing GP practice list sizes and sample distribution with the Scottish population.

Data quality, crucial for representativeness, was evaluated by sample rejection and form completion rates.

#### Timeliness

Timeliness reflects the speed or delay between steps in a surveillance programme [7, 8]. This was measured by adherence to deadlines and GP survey feedback.

#### Stability

The stability of a surveillance programme reflects its reliability to operate without failure [7, 8]. This was assessed by identifying breaks in data submission and unanalysed samples.

#### Usefulness

A public health surveillance system is useful if it aids in preventing and controlling adverse health events and improves understanding of their implications [7, 8]. Assessment was based on CARI data dissemination, scientific outputs, and public health decision-making.

#### Sensitivity and predictive value positive (PVP)

The sensitivity of a surveillance programme is evaluated by the proportion of cases detected, while the PVP is assessed by the proportion of identified cases that actually have the condition [7, 8]. Accurate assessment requires knowing the true number of cases, which is unavailable for CARI due to limited routine testing and the fact that many with respiratory symptoms do not seek medical care. Therefore, sensitivity and PVP could not be estimated for CARI.

### Statistical considerations

The total number of samples submitted, positive samples, and swab positivity (with 95% confidence interval [CI]) were determined over time, and stratified by age and sex. National Health Service (NHS) open data as of October 2023 [9] identified GP practice sizes, including by age and sex. Population estimates were from mid-2021 data published by the National Records of Scotland [10].

### Ethics

Ethical approval was not needed as the study was based on routine surveillance.

## Results

### Overall CARI findings

**Figure 2.**
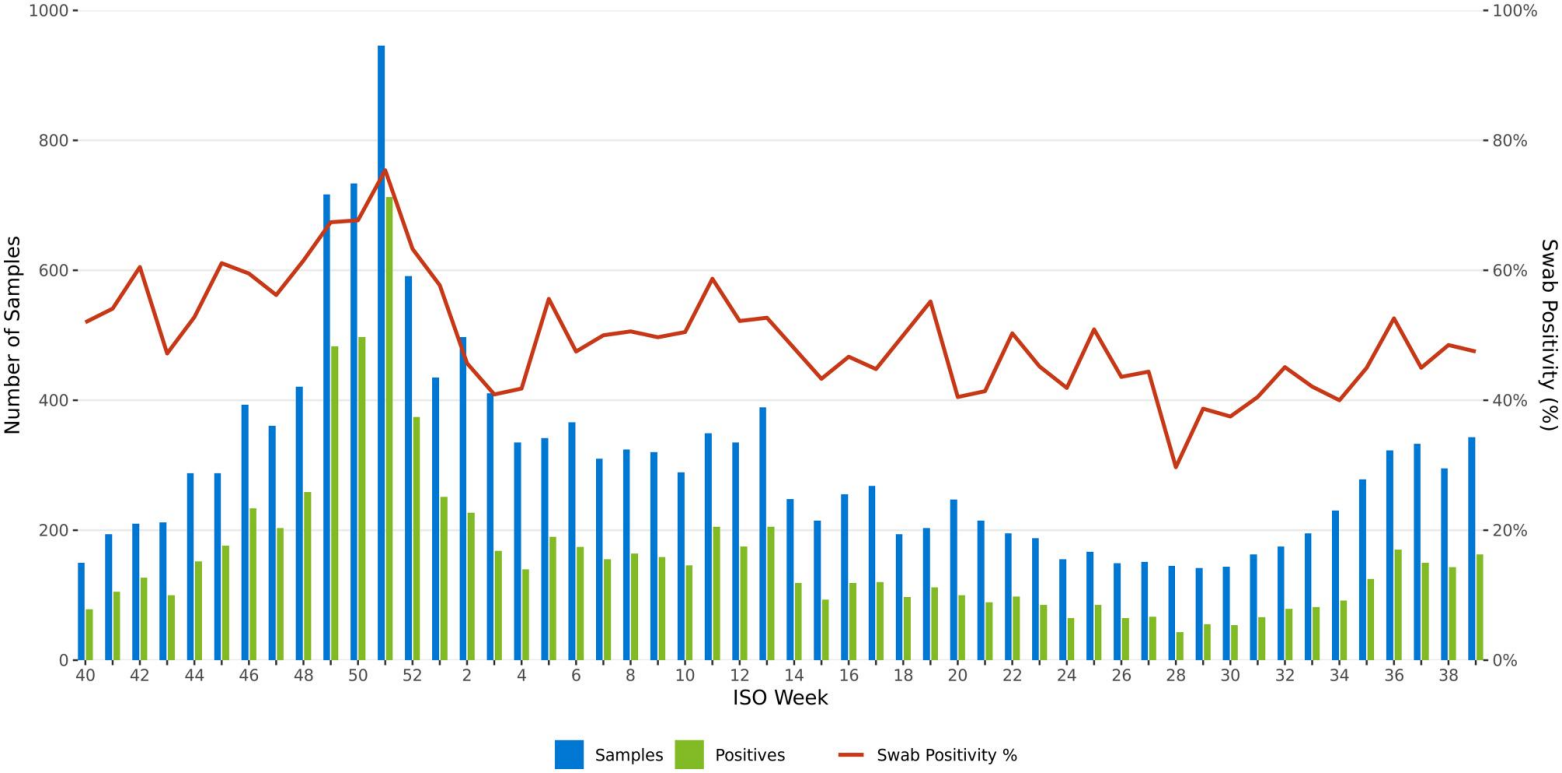
The number of samples, positive samples, and overall swab positivity over time, for the 2022/23 season.

During the study, 180 GP practices participated in CARI, testing 15 823 samples, detailing the ARI burden in the community. Of these, 1283 (8.1%) were self-swabbed. However, with only 32% of samples having ESFs and linked data, this limited further analyses. The actual proportion of self-swabbing may have been higher than reported. Figure 2 shows the number of samples, positive samples, and overall swab positivity over time. Sample submissions increased from week 40, peaking at over 900 in week 51, then declined to 200–300 per week. Overall swab positivity was 53.1% (95% CI: 52.3–53.8), with 56.5% (95% CI: 54.9–58.2) for GP-administered swabs and 53.9% (95% CI: 51.1–56.6) for self-swabbing. Overall swab positivity peaked between weeks 49 and 51, driven by influenza A.

**Figure 3.**
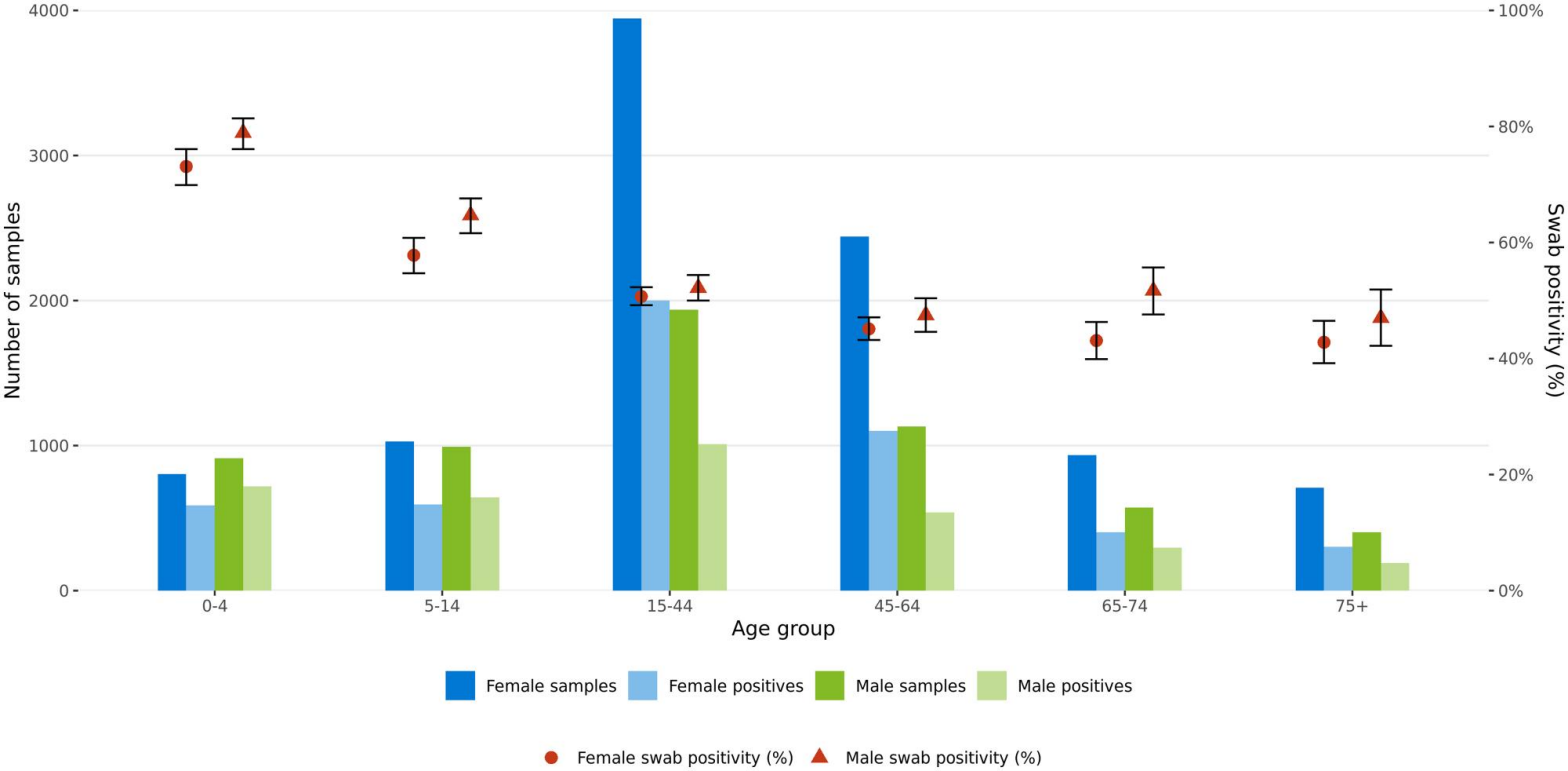
The number of samples, positive samples, and overall swab positivity, by age and sex, for the 2022/23 season.

Figure 3 shows the number of samples, positive samples, and overall swab positivity, by age and sex. Overall swab positivity varied, with the 0- to 4-year group showing the highest at 76.2% (95% CI: 74.1–78.1). Males exhibited higher positivity at 57.1% (95% CI: 55.8–58.3) compared to females at 50.6% (95% CI: 49.6–51.6).

### Pathogen-specific findings

**Figure 4.**
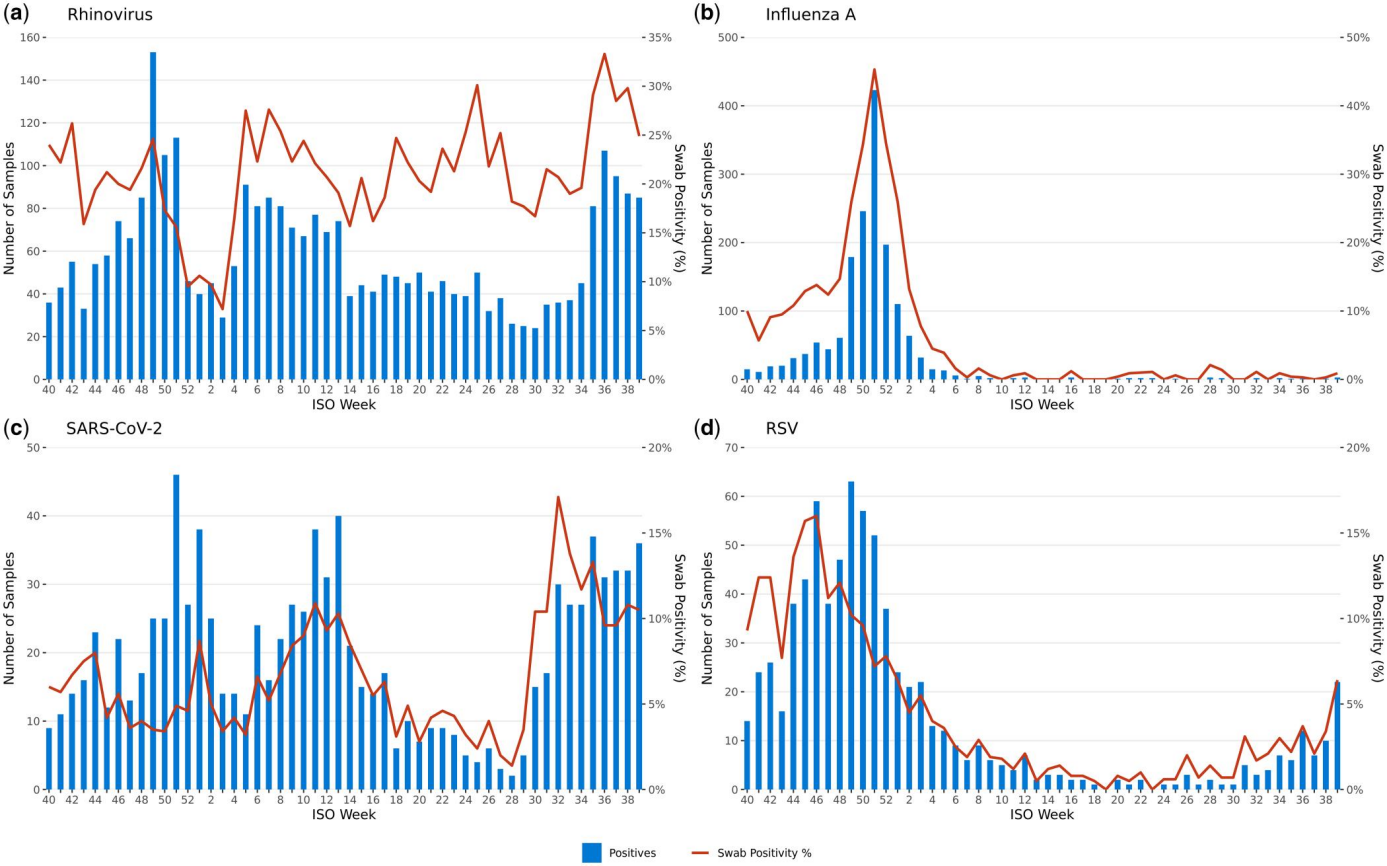
The number of positive samples and swab positivity over time for some of the key CARI pathogens, for the 2022/23 season.

Figure 4 presents the number of positive samples and swab positivity over time for some of the key CARI pathogens: rhinovirus, influenza A, SARS-CoV-2, and RSV. Rhinovirus was the predominant pathogen, with overall swab positivity at 20.5% (95% CI: 19.8–21.1), compared to 21.3% for the 2021/22 season. Peaks occurred in weeks 5, 7, 25, and 35–38, mirroring the previous season’s patterns.

Influenza A had the second highest overall swab positivity at 10.3% (95% CI: 9.9–10.8), compared to 2.7% the previous season. Swab positivity for H1, H3, and untyped was 3.1% (95% CI: 2.8–3.4), 5.6% (95% CI: 5.3–6.0), and 1.7% (95% CI: 1.5–1.9), respectively. Cases sharply increased between weeks 49 and 51, peaking at 45.3% in week 51.

Overall swab positivity for SARS-CoV-2 was 6.4% (95% CI: 6.0–6.8), compared to 10.2% for the 2021/22 season. Swab positivity fluctuated, with peaks early in the year, around spring, and in autumn. The highest recorded was 17.1% in week 32. Of 1011 positive samples, 696 were sequenced, revealing XBB.1.5 (*n* = 176), XBB (*n* = 127) and Omicron BQ.1 (*n* = 106) as the most frequent variants.

RSV positivity was 5.1% (95% CI: 4.7–5.4), compared to 4.5% the previous season. It peaked at 16.0% in week 46.

Detailed results from the CARI 2022/23 season, covering all the 10 CARI pathogens, are published elsewhere [11].

### Evaluation

#### Response to GP survey

Out of 87 responses, 78 were analysable after excluding nine blanks. As responses were anonymous, multiple submissions from the same practice were possible, making the response rate incalculable. Most respondents were GPs (55%, *n* = 43), with participation from practice managers, nurses, and other staff. Survey results were used to assess the surveillance system’s attributes.

#### Simplicity

The CARI swabbing kits, available for order via email, come in two types: clinician-swabbing or self-swabbing kits, each with tailored information. Survey feedback showed most respondents found it either easy or very easy to order (95.5%, *n* = 42/44), use (86.2%, *n* = 50/58), complete the virology request form (82.8%, *n* = 48/58) return samples (93.5%, *n* = 58/62), and access results (88.3%, *n* = 53/60). A small number faced challenges with ordering (2.3%, *n* = 1/44), completing forms (3.4%, *n* = 2/58), or returning samples (1.6%, *n* = 1/62). Main concerns were additional workload, especially with the online ESF, and result delays.

Diligent data management by dedicated team members with expertise in data processing and analyses contributed to simplicity of CARI by minimizing manual tasks.

#### Flexibility

Established during the COVID-19 peak, CARI demanded immediate adaptability in a shifting primary care context. For the 2022/23 season, simplified swabbing kits with prepaid return boxes were addressed issues from the previous GP survey. Information materials and data collection were also simplified.

GP survey results reflected positive feedback on CARI team responsiveness, with 95.1% (*n* = 39/41) who stated a preference agreeing their feedback was addressed. Furthermore, among respondents who provided a preference, 98.1% (*n* = 53/54) agreed or strongly agreed that collaborating with the CARI team was straightforward.

#### Acceptability

Throughout the study, 180 GP practices participated in CARI, with 16 withdrawing mid-season. Practices do not need to give a reason for why they want to withdraw, but a small number did mention pressure of work as being a factor. Despite this, 92.3% (*n* = 72/78) of survey respondents were satisfied with their participation, and 77.4% (*n* = 48/62) were satisfied with sample reimbursement. Additionally, 91.7% (*n* = 55/60) would advocate for CARI, and 81.7% (*n* = 49/60) noted benefits like increased infection awareness and improved patient advice against unnecessary antibiotics. However, some respondents noted complexities and time constraints in the swabbing process. Dissatisfaction with reimbursement was reported by 1.6% (*n* = 1/62), and 2.6% (*n* = 2/78) were overall dissatisfied. Only 3.3% (*n* = 2/60) would not endorse participation, and 3.3% (*n* = 2/60) perceived no benefit.

#### Representativeness

##### Geographical representativeness

The geographic distribution of the Scottish population across 14 health boards was compared to the population served by CARI GP practices within each health board (Supplementary Fig. S1). GP practices signed up to CARI comprised 26.0% of the Scottish population, varying by health board from 10.8% in Forth Valley to 55.9% in Borders.

The proportion of all CARI samples received from each health board in the 2022/23 season was compared to each board’s Scottish population share (Supplementary Fig. S1). Some boards contributed disproportionately to their population share, such as Ayrshire and Arran (6.7% of the population, 17.7% of samples), Borders (2.1% of the population, 6.0% of samples) and Grampian (10.7% of the population, 5.7% of samples). Larger boards had contributions proportional to their populations: Greater Glasgow and Clyde (21.6% of the population, 22.9% of samples), Lothian (16.7% of the population, 15.4% of samples), and Lanarkshire (12.1% of the population, 14.4% of samples).

##### Demographic representativeness

The age distribution served by CARI GP practices was compared to the overall population within each health board (Supplementary Fig. S2). There were some variations in the age distribution served by CARI GP practices compared to the overall population within each health board. For example, the 0–4 age group made up 4.7% of Scotland’s population, varying from 3.9% in Western Isles to 5.0% in Lanarkshire. Within CARI practices, this age group was 3.9%, ranging from 3.5% in Greater Glasgow and Clyde and Western Isles to 5.2% in Orkney. There were also minor discrepancies in the gender distribution, with women representing 50.9% of CARI patients (data not shown), versus 51.2% in the general Scottish population.

#### Data quality

Data quality, essential for representativeness, depends on completeness and validity [7, 8]. Throughout the study, 3.3% (523/15 714) of samples were rejected, primarily due to incomplete patient details on the sample (73.2%, *n* = 383/523) or missing request form details (6.5%, *n* = 34/523). Further on completeness, only 32.0% of the samples had an ESF completed, with just over half of these (55%) completed by the patient.

#### Timeliness

Most CARI sample results, tested by Sunday, were publicly available the following Thursday.

Overall, CARI has demonstrated good timeliness, with prompt sample processing and result dissemination, although some GP survey respondents felt there were delays in receiving individual patient test results.

#### Stability

The effectiveness of CARI depends on seamless data flow, from recruitment and swabbing to laboratory processing and result publication. During the 2022/23 season, technical issues in the laboratory and postal strikes caused significant delays, particularly from December 2022 to January 2023. On average, 45.6% of swabs were not fully processed for all 10 pathogens by Thursday, impacting data analysis and publication. This issue peaked at 83.8%–99.8% between week 48, 2022 and week 2, 2023.

The number of participating GP practices in CARI increased from 90 to 164 during the 2022/23 season. While this growth may have strained laboratory capacity and affected sample testing stability, it otherwise strengthened the robustness of CARI analyses.

#### Usefulness

The CARI programme provided continuous respiratory surveillance data for real-time monitoring and public health decision-making. Weekly results on overall and pathogen-specific swab positivity, along with symptom data, provided a comprehensive understanding of Scotland’s respiratory disease burden. Internal and external dissemination to PHS, government, health protection teams, and GP practices ensured widespread respiratory situational awareness. Ad hoc scientific analyses integrated CARI data with other sources, notably contributing to a Chief Medical Officer (CMO) letter in November 2022 [12], on antiviral use for seasonal influenza.

## Discussion

Following the COVID-19 pandemic, uncertainties about pathogen prevalence shifts led WHO to emphasize the importance of integrated respiratory surveillance [1]. PHS was successful in establishing an integrated, comprehensive respiratory surveillance system for 10 respiratory pathogens for the 2022/23 season. The CARI sentinel surveillance programme consistently provided data, revealing a peak in influenza A in weeks 51–52, variable SARS-CoV-2 activity, and the prevalence of common pathogens like rhinovirus. It effectively met its overall objective of monitoring the burden and impact of ARI in the community.

Surveillance programmes rely on the active stakeholder involvement, and CARI excelled in this area with high GP engagement, resulting in high numbers of sample submission rates. Key factors included simplified processes that clinicians found easy to follow, attractive incentives, and additional benefits beyond surveillance. Notably, CARI emphasized relationship-building and collaboration through regular meetings with sentinel sites, requests for feedback with prompt responses, and weekly sharing of results. We strongly recommend implementing robust stakeholder engagement strategies when establishing new sentinel surveillance systems.

Patient participation is integral to effective surveillance. A limitation of the CARI programme is the lack of data on the number of eligible patients who declined swabbing, were offered self-swabbing kits, or did not return kits. Despite this, GPs reported manageable recruitment, indicating general acceptance of the programme. However, only 32% of patients had the ESF completed (by patient or clinician) in the 2022/23 season, suggesting possible logistic issues such as accessibility, time constraints, or perceived relevance. Additionally, some GPs felt the ESF was burdensome or daunting for patients, and the introduction of a separate online link added complexity to the process.

Acknowledging the significance of patient involvement, an upcoming patient survey aims to explore patient-centric aspects of CARI. This initiative reflects a commitment to improving patient participation and refining the programme’s effectiveness.

To maximize the utility of surveillance data, a programme must ensure geographic and demographic representation. Although CARI GP practices covered a considerable portion of the Scottish population, regional differences emerged. Forth Valley (10.8%) and Highland (14.8%) fell below the 15% coverage target [5]. Certain health boards contributed disproportionately, possibly due to differences in GP swabbing practices, or regional illness prevalence. Recognizing these disparities can guide targeted recruitment and communication strategies to enhance programme representativeness. Additionally, variations across age groups and gender were observed, potentially influenced by pathogen distribution, GP visit thresholds, and testing willingness. Further investigation into these complexities will inform strategies for improved programme inclusivity and effectiveness.

Effective data management is crucial for maintaining data quality and completeness. The CARI team, composed of trained experts in data processing, analysis, epidemiology, and public health, meticulously oversees all data management tasks. Their dedicated efforts ensure thoroughness and accuracy throughout the entire process.

The high quality of CARI data was demonstrated by its consistency with signals from secondary care and across Europe. During the 2022/23 season, CARI detected a notable surge in influenza A swab positivity, peaking at 45.3% in week 51. This peak coincided with Scotland’s highest influenza hospital admission rates since 2016/17 and a similar trend observed across Europe [13, 14]. Additionally, RSV swab positivity increased compared to the previous season, reflecting a Europe-wide pattern of an earlier and more severe RSV season, which put considerable strain on healthcare systems with increased paediatric admissions [15].

Real-time surveillance enables rapid public health interventions. Aggregated surveillance data was published on a Thursday, covering the preceding week, although scientists had earlier oversight of these data. This schedule aligns with UK Health Security Agency timelines [16]. However, operational stability and timely data processing posed challenges, particularly during periods of technical disruptions and logistical issues. Implementing strategies to mitigate delays in sample processing are crucial for uninterrupted data flow.

The success of a surveillance programme depends on its ability to fulfil crucial public health functions and optimize resource allocation. Striking this balance necessitates integrating key attributes to ensure the programme’s overall quality [7, 8]. Programmes that are simple, flexible, acceptable, and stable are better positioned to facilitate effective public health action. The CARI programme has demonstrated achievement in these regards, performing well across these attributes. Additionally, CARI data play a vital role in informing key decision-makers about trends that may signal changes in the occurrence of respiratory disease, enabling timely public health responses.

### Limitations

As with any surveillance programme, CARI data may not fully capture the landscape of respiratory pathogens in the community setting due to the ARI case definition’s sensitivity and specificity constraints. Incidence rates for each pathogen could not be calculated due to unknown proportions of patients fitting the CARI case definition who were not recruited and swabbed. Additionally, the proportion of patients with enhanced surveillance data was low.

The GP survey responses may not fully represent the diversity of participating practices, and multiple submissions from staff within the same practice could introduce bias. Furthermore, the lack of data on Scottish Index of Multiple Deprivation (SIMD) [17] limited demographic assessment. While efforts to collect patient feedback are underway, evaluation of patient perspectives on the CARI programme was not feasible within the scope of this study.

## Conclusion

The study highlights both the effectiveness and challenges of the CARI programme during the 2022/23 season. The programme provided valuable insights into respiratory pathogen prevalence and seasonal trends, reflecting patterns observed in secondary care and across Europe. As a cornerstone of Scotland’s infectious respiratory diseases plan, the programme’s ability to capture community signals underscores its significance. While effective, ongoing efforts are crucial for maximizing CARI’s public health impact.

This evaluation highlights two key areas for improvement: geographical representativeness and enhanced surveillance data collection. To address these, a targeted recruitment strategy will aim to increase participation in underserved areas, and a simplified process for collecting enhanced surveillance data has been developed. This new approach requires GPs or patients (for self-swabbing) to provide symptom data on the same form as the CARI test request, thereby reducing the number of steps in the CARI process. While the CARI programme is not yet fully digital, there is a commitment to continued improvement, with a long-term goal of full automation and digitalization.

## Supplementary Material

ckae200_Supplementary_Data

## Data Availability

The data underlying this article cannot be shared publicly due to the privacy of individuals who participated in the study. Key points The study found that the CARI surveillance programme is a crucial and effective tool for tracking respiratory infections in communities across Scotland. By effectively detecting and monitoring respiratory infection trends, CARI can help public health authorities respond quickly with targeted actions to control the spread of diseases. The evaluation of CARI’s performance highlights its successes and areas for improvement, providing feedback that can enhance its effectiveness as a valuable tool for protecting public health in Scotland. Additionally, the evaluation offers valuable insights for surveillance systems beyond Scotland, highlighting key lessons that, if integrated, can enhance other programmes’ ability to proactively monitor and manage respiratory infections, thereby protecting public health in their regions.
