# Supplementary figures and images for: The Community Acute Respiratory Infection surveillance programme: an evaluation of a newly established surveillance programme in Scotland
Source: Eur J Public Health. 2024 Nov 28;35(2):282–9. doi: 10.1093/eurpub/ckae200 (PMC11967905; doi:10.1093/eurpub/ckae200)

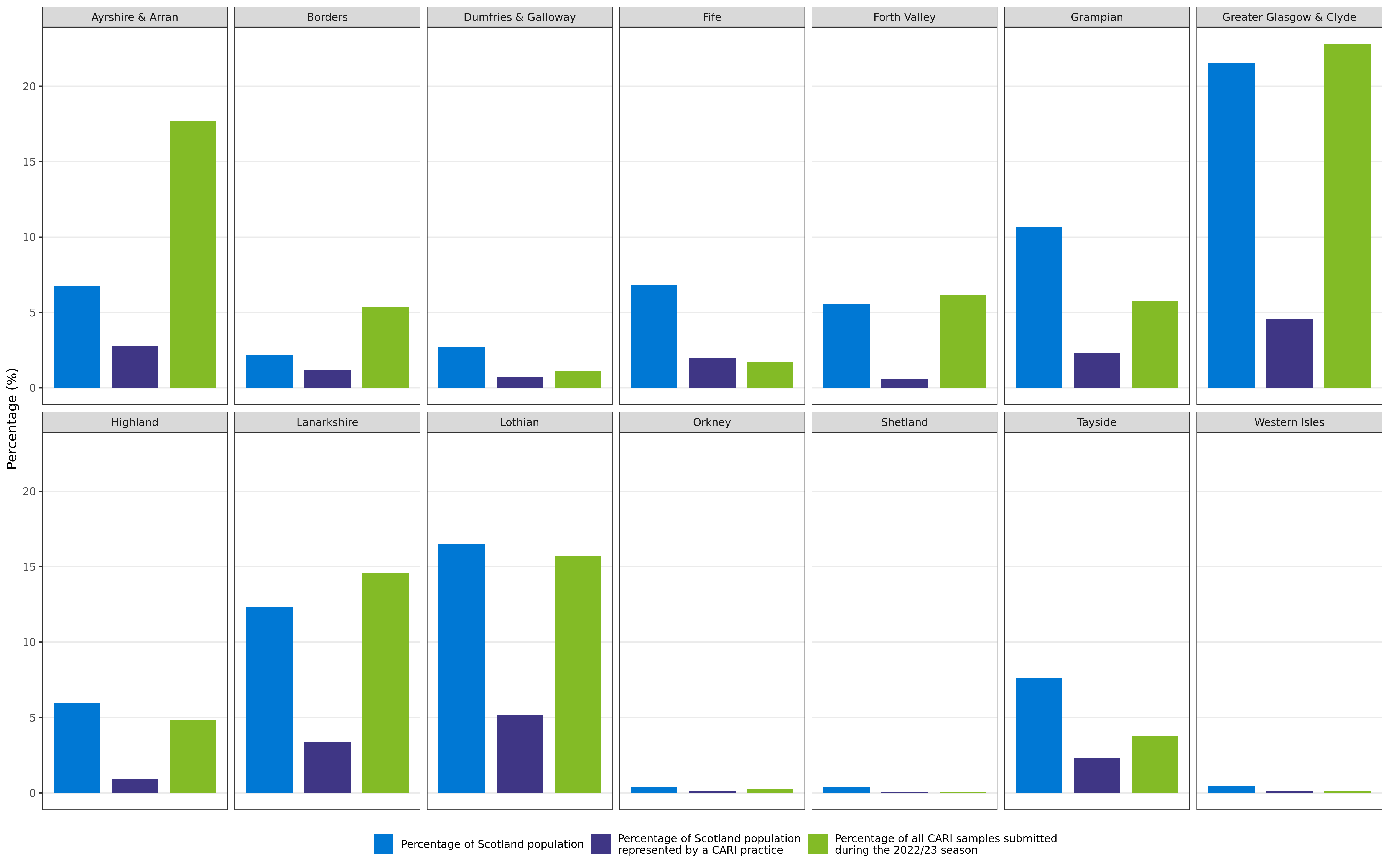

Supplement: ckae200_Supplementary_Data [file ckae200_supplementary_data.zip › ckae200_Supplementary_Data/ejph-2024-07-om-0472-File006.tif]

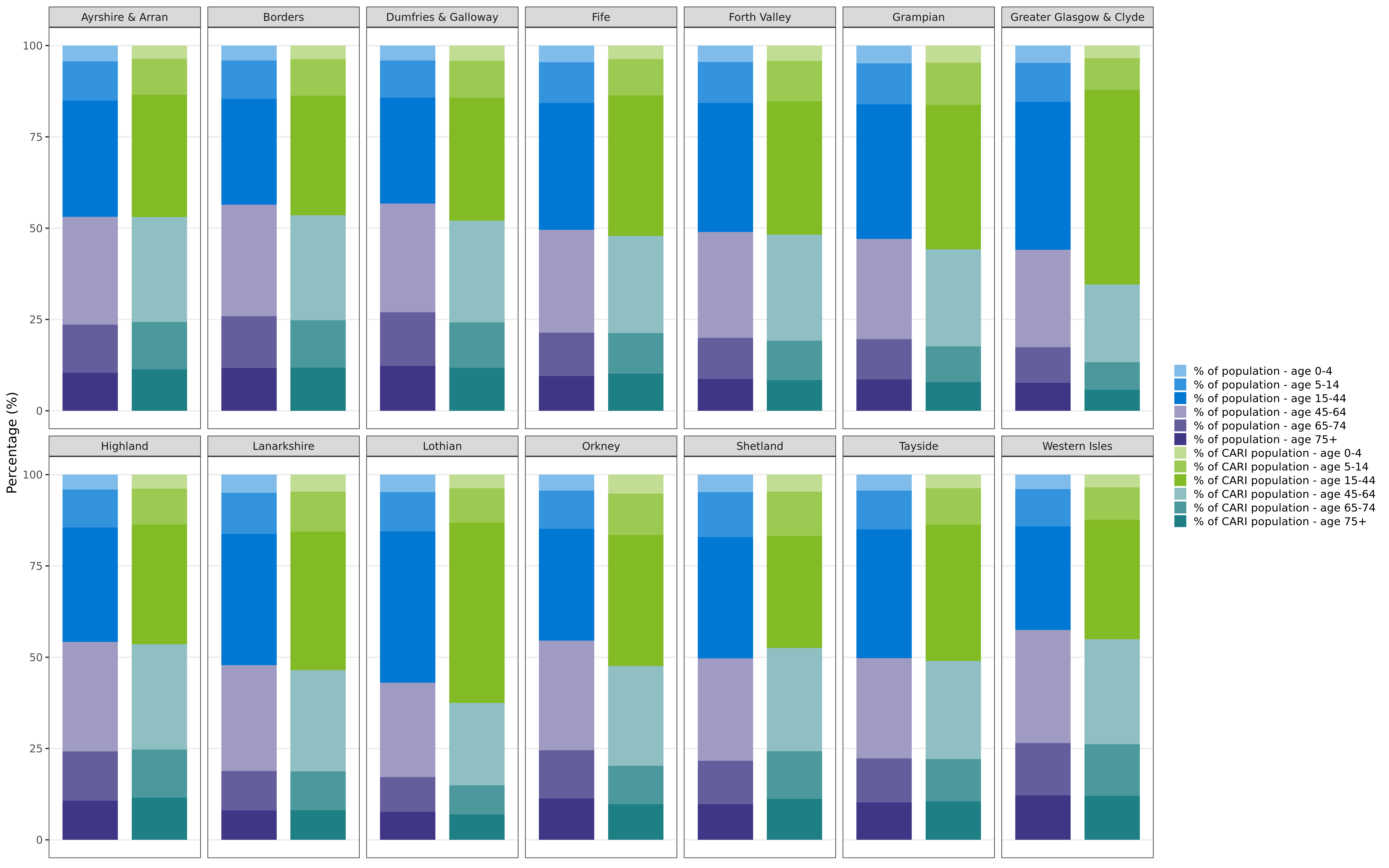

Supplement: ckae200_Supplementary_Data [file ckae200_supplementary_data.zip › ckae200_Supplementary_Data/ejph-2024-07-om-0472-File007.tif]
